# Compounding effects in flood drivers challenge estimates of extreme river floods

**DOI:** 10.1126/sciadv.adl4005

**Published:** 2024-03-27

**Authors:** Shijie Jiang, Larisa Tarasova, Guo Yu, Jakob Zscheischler

**Affiliations:** ^1^ Department of Compound Environmental Risks, Helmholtz Centre for Environmental Research, Leipzig, Germany.; ^2^ Department of Biogeochemical Integration, Max Planck Institute for Biogeochemistry, Jena, Germany.; ^3^ ELLIS Unit Jena, Jena, Germany.; ^4^ Department Catchment Hydrology, Helmholtz Centre for Environmental Research, Halle (Saale), Germany.; ^5^ Division of Hydrologic Sciences, Desert Research Institute, Las Vegas, NV, USA.; ^6^ Technische Universität Dresden, Dresden, Germany.; ^7^ Center for Scalable Data Analytics and Artificial Intelligence (ScaDS.AI), Dresden-Leipzig, Germany.

## Abstract

Estimating river flood risks under climate change is challenging, largely due to the interacting and combined influences of various flood-generating drivers. However, a more detailed quantitative analysis of such compounding effects and the implications of their interplay remains underexplored on a large scale. Here, we use explainable machine learning to disentangle compounding effects between drivers and quantify their importance for different flood magnitudes across thousands of catchments worldwide. Our findings demonstrate the ubiquity of compounding effects in many floods. Their importance often increases with flood magnitude, but the strength of this increase varies on the basis of catchment conditions. Traditional flood analysis might underestimate extreme flood hazards in catchments where the contribution of compounding effects strongly varies with flood magnitude. Overall, our study highlights the need to carefully incorporate compounding effects in flood risk assessment to improve estimates of extreme floods.

## Teaser

The growing importance of compounding effects in larger floods can complicate estimates of extreme flood hazards.

## INTRODUCTION

River floods are among the most common natural disasters, and their risk is projected to increase further in the future due to climate and socioeconomic changes, although substantial uncertainties remain (*1*, *2*). Key to improving flood risk analysis is an improved scientific understanding of the mechanisms that lead to floods, especially those associated with extreme floods (*3*–*9*). River floods can be generated by a variety of atmospheric processes (e.g., circulation patterns causing heavy precipitation and temperature increases causing snowmelt or glacial melt) that are modulated by the conditions and characteristics of the catchment (*10*). Complex interactions between all of these factors determine the timing, duration, extent, temporal clustering, and severity of river floods (*11*–*14*), which makes estimating future flood risks particularly challenging because flood drivers may exhibit varying trends in a changing climate (*15*, *16*).

Although it is recognized that river floods are typically affected by multiple drivers (*5*–*7*, *11*, *13*, *16*–*18*), a quantitative and systematic analysis that disentangles the interaction effects between these drivers and their implications for flood generation remains underexplored, especially at a large scale and event-specific level. These are often particularly important for extreme flood events due to potential amplifying effects between different physical processes across various spatial and temporal scales (here referred to as compounding effects) (*19*, *20*). A thorough understanding of the compounding effects between river flood drivers under historical conditions is critical for improving current flood risk adaptation strategies and projecting future flood risks (*3*, *21*). Moreover, the importance of compounding effects may vary depending on the magnitude of the flood and the specific catchment (*22*, *23*), which is not well understood. In certain catchments, the importance of these effects may exhibit considerable variability across flood magnitude, while, in others, the differences may be negligible. A large variability would imply heterogeneity in flood-generating processes and challenge the reliability of conventional statistical methods used to estimate extreme flood hazards, which typically rely on an assumption of homogeneity (*24*–*26*). It is therefore crucial to identify the catchment-specific variability in compounding effects, which can further be used to assess potential errors associated with extreme flood estimation. This task requires effectively capturing the nonlinear interactions between flood drivers and the quantification of their joint contributions at the event scale.

**Fig. 1.**
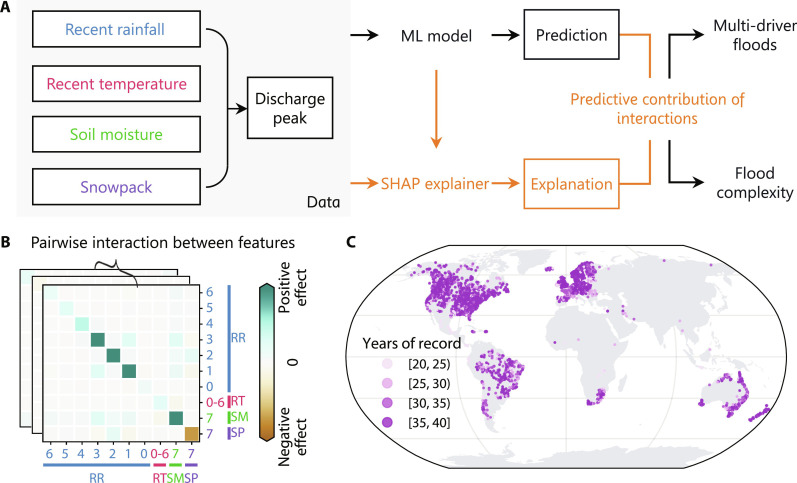
Procedure for identifying compounding effects in river flood drivers. (**A**) Conceptual diagram of the methodological framework. Meteorological drivers, including recent rainfall (RR) and recent temperature (RT), and catchment preconditions, including soil moisture (SM) and snowpack (SP), are used as input variables. The model output refers to all identifiable discharge peaks regardless of their magnitude (fig. S2). The interpreted interaction effects are used to identify multi-driver floods associated with compounding drivers and to determine flood complexity in subsequent analyses. (**B**) Illustration of SHAP interaction values for machine learning (ML) model outputs for exemplary discharge peaks. The tick labels indicate the different types of variables, distinguished by different colors as indicated in (A). The number represents the number of days before a discharge peak for the corresponding variable. (**C**) Locations of the 3527 catchments used in this study and the length of discharge records in individual catchments.

In this study, we developed an approach based on advanced explainable machine learning (ML) to disentangle the importance of compounding effects in river floods (Fig. 1A). The approach combines light gradient boosting machine (LightGBM) (*27*) for the prediction of runoff events and Shapley additive explanation (SHAP) interaction values (*28*) to estimate the predictive contributions of input features and their interactions. Here, SHAP interaction values are used to assess the contribution of each pair of features to the prediction outcome in our ML model by identifying both the main effects of individual features and the pairwise interaction effects between features. This framework provides a unified approach to quantifying how interactions between meteorological drivers (rainfall and temperature) and catchment preconditions (snow depth and soil moisture) (fig. S1) may influence river floods (defined as annual maximum discharges) (*12*, *13*) for a given catchment (Fig. 1B). We applied this approach to 3527 catchments worldwide (Fig. 1C) and identified compounding drivers and the role of compounding effects for 124,642 annual discharge maxima from 1981 to 2020 (Materials and Methods). Moreover, we quantified the catchment-specific variability in the importance of compounding effects across different flood magnitudes. This catchment property, which we term “flood complexity,” characterizes the heterogeneity in the physical processes underlying floods of varying magnitudes in individual catchments. In the study, we address the following questions: (i) How prevalent are compounding drivers in historical river floods, and what impacts do they have on flood severity? (ii) How does the relationship between the importance of compounding effects and flood magnitude (i.e., flood complexity) vary under different catchment conditions? (iii) What are the implications of high flood complexity for the reliability of flood hazard estimates? Overall, the study aims to advance our understanding of how compounding flood drivers might question conventional estimates of extreme floods, underlining the necessity for tailored, catchment-specific strategies in flood mitigation design.

## RESULTS

### Spatial distribution of multi-driver floods and association with flood severity

We first investigated the spatial distribution of flood events associated with compounding drivers (i.e., multi-driver floods) and their association with flood severity. We aggregated the SHAP interaction values additively (*28*) to estimate the total respective contribution of recent rainfall, recent temperature, soil moisture, and snowpack (variables are color-coded in Fig. 1B). To identify candidates for the main driving variables of each discharge peak, we defined a threshold at the 80th percentile of all aggregated SHAP values for an individual catchment (fig. S4). To ensure the robustness of the results, we used an ensemble of ML models trained on different bootstrapped subsets of the data, allowing each peak sample to be evaluated 100 times by different models. We further used a binomial test to determine the main driving variables from the candidate variables identified by these evaluations. Multi-driver floods are defined as those that consistently exhibited an association with more than one candidate variable across numerous evaluations (refer to Materials and Methods for more details).

**Fig. 2.**
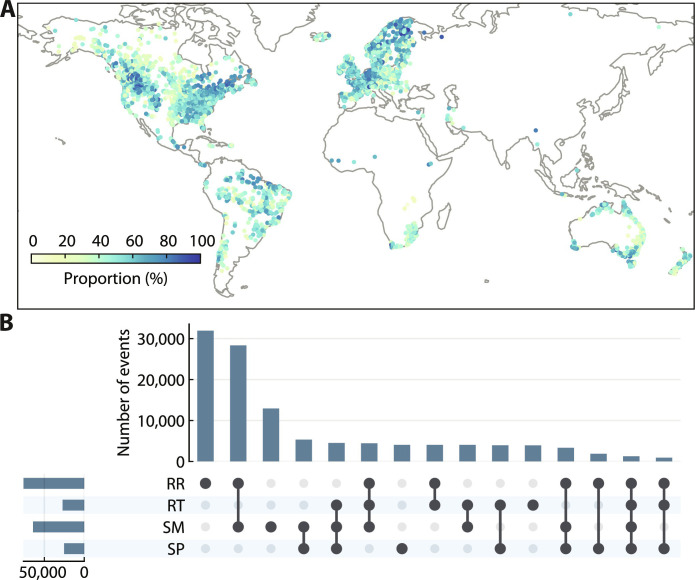
Multi-driver flood events in the 3527 catchments. (**A**) Proportion of flood events associated with compounding drivers. (**B**) Upset plot comparing the combinations of the four main driving variables: RR, RT, SM, and SP. The horizontal bars on the bottom left denote the number of flood events associated with each variable. Each unique combination of variables is represented by a line connecting the filled-in cells, while the height of the vertical bars signifies the number of events presenting that specific combination. The 80th percentile of the aggregated contributions of all samples in each evaluation was used as the cutoff for determining main driving variables and identifying multi-driver events (see Materials and Methods).

For all identified flood events, 61.1, 21.8, 51.5, and 20.3% are associated with recent rainfall, recent temperature, soil moisture, and snowpack, respectively. The main variables influencing the annual maximum discharge events vary considerably between catchments (fig. S5), demonstrating spatial heterogeneity in the flood-generating processes (e.g., precipitation, soil moisture saturation, and snowmelt). In addition, the subtle role of temperature in non-snowmelt regions may indirectly indicate the influence of factors such as evapotranspiration. The spatial patterns of the driving variables are largely consistent with previously identified global and regional flood types in the literature (*3*, *6*, *9*), which typically assign one dominant process to each flood event, but our emphasis lies in quantifying the specific contribution of individual drivers. Figure 2A reveals the distribution of multi-driver floods that are associated with at least two of the four main variables identified above. Of the 124,642 flood events analyzed, 51.6% are associated with at least two driving variables. Almost all the studied catchments have experienced multi-driver floods to a greater or lesser extent. In 55.1% of the catchments, more than 50% of the floods are multi-driver floods. The variety of identified driver combinations suggests the existence of complex interplays between precipitation, temperature, antecedent soil moisture, and snowpack in flood generation (Fig. 2B), emphasizing the importance of understanding river flood risks from a multivariate perspective (*21*). In particular, the joint contribution of recent rainfall and soil moisture accounts for the largest proportion of multi-driver floods, which highlights the preconditioning role of antecedent soil moisture in flood development (*6*, *29*).

**Fig. 3.**
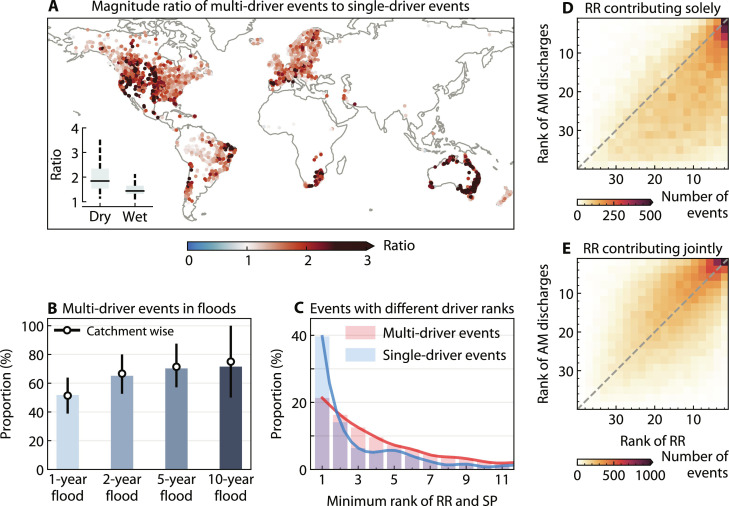
Impact of compounding drivers on the severity of river flood events. (**A**) The magnitude ratio of multi-driver floods to single-driver floods in individual catchments. The ratio was calculated as the mean magnitude of multi-driver floods divided by the mean magnitude of single-driver floods. The inset box plot compares the magnitude ratio between dry catchments (CMI < 0) and wet catchments (CMI ≥ 0) (fig. S3A). (**B**) Proportion of multi-driver floods associated with different return periods. The height of the bars indicates the event-wise proportion, whereas the points indicate the median of the catchment-wise proportions and error bars indicate the 25th and 75th percentiles. (**C**) Comparison between multi-driver and single-driver floods in terms of the minimum rank of RR and antecedent SP before individual events. The minimum rank is determined on the basis of the lower of the catchment-specific ranks of RR and SP for the largest flood event in each catchment. Lower ranks correspond to higher magnitudes. (**D** and **E**) Event-wise ranks of RR against event-wise ranks of annual maximum (AM) discharges when RR is the sole contributor (D) and when RR contributes in combination with other drivers (E). The color indicates the two-dimensional histogram of RR-AM discharge rank pairs across all catchments, with darker shades indicating more counts. The number of bins for each dimension was set to 20.

To evaluate the impact of compounding drivers on flood severity, we compared the mean magnitude of multi-driver floods and single-driver floods in individual catchments (Fig. 3A). We find that the mean magnitude of multi-driver floods is significantly higher than that of single-driver floods in 56.1% of the 3527 catchments (one-sided *t* test, α *=* 0.05). Among these catchments, the mean magnitude of multi-driver floods is at least 20% higher than that of single-driver floods in 63.9% of catchments and at least 50% higher in 28.5% of catchments. The catchments with a higher magnitude ratio are generally characterized by a high degree of aridity (fig. S3A), with a strong negative correlation between magnitude ratio and the climate moisture index (CMI) of the catchments (Spearman’s rank correlation coefficient = −0.55, *P* < 0.001). Previous studies have consistently found that drier catchments have heavier flood tails (*26*, *30*), and our results suggest that compounding drivers that tend to increase nonlinear interactions between processes may be a contributing mechanism.

Moreover, the likelihood of floods being associated with multiple drivers generally increases as floods become more extreme (Fig. 3B). The proportion of multi-driver events in all annual maximum floods (124,642 events in total) increased from 51.6 to 64.9, 70.1, and 71.3% for floods with a catchment-specific return period of at least 2 years (63,199 events), 5 years (24,289 events), and 10 years (11,338 events), respectively. The catchment-wise proportions largely follow the same pattern across different return periods (Fig. 3B), with significant differences as confirmed by both the one-sided Wilcoxon rank sum test and one-sided Wilcoxon signed-rank test (*P* < 0.001). Notably, the variance of these proportions increases for 10-year floods. In some catchments, multi-driver floods may be less dominant for extreme floods than for moderate floods (e.g., 5-year floods), particularly when a single driver is capable of exerting a sufficiently dominant influence on the generation of extreme floods (*16*).

We further compared the extremeness of the drivers between the largest single-driver and multi-driver floods globally from 1981 to 2020 (Fig. 3C), where rainfall and snowpack are collectively accounted for to accommodate the diversity of flood types. The largest single-driver floods tend to exhibit the most extreme event-wise rainfall or snowpack. In contrast, the extremeness of rainfall or snowpack that triggers multi-driver floods is more widely distributed, suggesting that non-extreme drivers can, nevertheless, drive extreme outcomes. A comparison of the relevance of the event-wise rank of the recent rainfall and flood magnitude in each catchment further reveals differences between floods induced by recent rainfall only and floods jointly triggered by recent rainfall and additional drivers. When recent rainfall contributes to flooding events in combination with other drivers, the mean rank of recent rainfall is significantly higher than that of the annual maximum flood (Fig. 3E; one-sided paired *t* test, *P* < 0.001). In contrast, when recent rainfall is the single flood driver, even extreme recent rainfall does not always cause large floods (high density below the diagonal in Fig. 3D), which is likely due to buffering effects (i.e., negative contributions) from other drivers (e.g., drier soils). Limiting the analysis to catchments with a longer observation period (e.g., at least 35 annual maximum discharges) does not change the above conclusion. These combined results demonstrate again the role of compounding drivers in amplifying the flood magnitude of a river and illustrate that extreme outcomes can result from moderate drivers, which underscores the importance of considering the compounding nature of flood drivers in risk management.

Because the identification of main driving variables is highly dependent on the selected threshold, we conducted a number of sensitivity tests. Generally, lower thresholds lead to a higher number of contributing drivers, resulting in more flood events being considered as multi-driver floods, and vice versa for higher thresholds. However, different thresholds lead to similar spatial patterns of multi-driver flood proportions (fig. S6), showing the robustness of our conclusions against different threshold choices. In particular, the proportions of multi-driver floods using different thresholds are strongly correlated. The conclusions about the relevance of compounding drivers and flood severity drawn in Fig. 3 hold with different thresholds as well (figs. S7 and S8). In addition, a stricter criterion for the predictive performance of the ML algorithms does not affect the above conclusion (fig. S9).

### Flood complexity: Variability of compounding effects with flood size

**Fig. 4.**
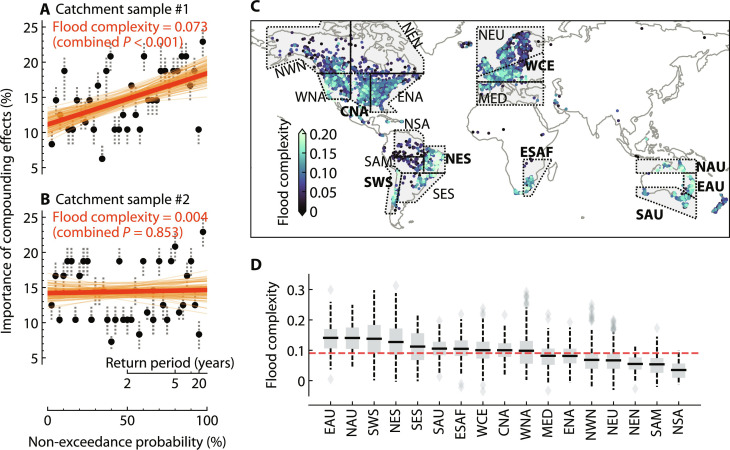
Comparison of flood complexity across catchments. (**A** and **B**) Illustrative examples of the flood complexity of two catchments, one located in Slovakia with an outlet at 49.07°N, 18.91°E (A) and the other in the United Kingdom with an outlet at 54.44°N, 3.53°W (B). Points denote the median of the compounding effects importances over the 100 evaluations, with the error bars indicating the 25th and 75th percentiles. The importance of compounding effects (exemplified in fig. S10) is based on the number of interaction values exceeding a threshold (here, the 80th percentile of the positive interaction values across all samples). The dashed lines indicate the fitted slope in each of the evaluations, and the flood complexity is the median of these slopes (the solid orange line). The combined *P* value is estimated using Fisher’s method from individual *P* values that indicate the significance of whether the corresponding slope is positive. (**C**) Spatial distribution of flood complexity for individual catchments (median across 100 evaluations). The gray polygons represent the IPCC climate reference regions (*66*), each of which contains at least 50 study catchments. The bold abbreviation indicates the region has an average flood complexity significantly higher than the global average (one-sided *t* test, α = 0.001). (**D**) Flood complexity of catchments in IPCC reference regions. The red dashed line indicates the average flood complexity across all catchments. Box plots show the median, 25th percentile, 75th percentile, and 1.5× interquartile range of the data. EAU, eastern Australia; NAU, northern Australia; SWS, southwestern South America; NES, northeastern South America; SES, southeastern South America; SAU, southern Australia; ESAF, east southern Africa; WCE, western and central Europe; CNA, central North America; WNA, western North America; MED, Mediterranean; ENA, eastern North America; NWN: northwestern North America; NEU, northern Europe; NEN, northeastern North America; SAM, South American monsoon; NSA, northern South America.

As suggested by Fig. 3, the impact of compounding drivers on flood magnitude varies across catchments. A given catchment may also exhibit substantial variability in the importance of compounding effects between different events, indicating a high degree of catchment-specific complexity in terms of flood generation processes. In this case, insights gained from smaller floods may not be applicable when estimating the magnitude of larger events. To assess the flood complexity of a given catchment, we used the simple linear regression slope between the importance of compounding effects and the empirical non-exceedance probability of flood events. Here, the importance of compounding effects for individual flood events is defined as the relative number of main interaction effects (by thresholding the SHAP interaction values) that have a large and positive contribution to increasing discharge (fig. S10). In contrast to the previous section, we now consider all 48 possible interactions. Flood complexity can serve as an indicator of flood process heterogeneity resulting from compounding effects between drivers. This is illustrated in Fig. 4 (A and B) that shows the flood complexity of two different catchments; it is evident that the flood generation in the Slovakian catchment (Fig. 4A) exhibits greater heterogeneity compared to the British catchment (Fig. 4B).

We estimated flood complexity for all 3527 catchments (Fig. 4C). Of the catchments studied, 96.1% have a significantly positive slope (combined *P* < 0.01 by Fisher’s method from the 100 evaluations). Catchments with a low flood complexity (often coinciding with nonsignificant flood complexity overall) are mainly found in the northern regions, the Alpine region, and the Amazon Basin. For high-latitude and high-altitude regions, flood generation tends to be uniformly dominated by snowmelt, and, therefore, the importance of compounding effects is likely to be homogeneous across flood magnitudes. In contrast, floods in the Amazon Basin are typically triggered by saturated soil moisture that has accumulated throughout the rainy season (*31*), resulting in low variability in the number of driver interactions during the few days preceding different magnitudes of flood events.

Regions with average flood complexity of catchments significantly higher than the global average (one-sided *t* test, *P* < 0.001) mainly include eastern Brazil, the Andes, eastern Australia, the Rocky Mountains extending to the west coast, and the western and central European plains (Fig. 4, C and D). Catchments in these regions typically have multiple flooding mechanisms. For example, catchments in the European plains may experience flooding caused by recent rainfall alone or by both recent rainfall and snowmelt/antecedent soil moisture (*5*, *9*). The various combinations of factors and processes involved in the generation of the catchment response produce a wide range of hydrologic behaviors with varying degrees of interactions and nonlinearity (*32*). The estimated flood complexity is not dependent on the choice of the threshold that determines the main interaction effects, which demonstrates the robustness of the above results (fig. S11, A to D).

### Relation to catchment attributes and implications for estimating large floods

**Fig. 5.**
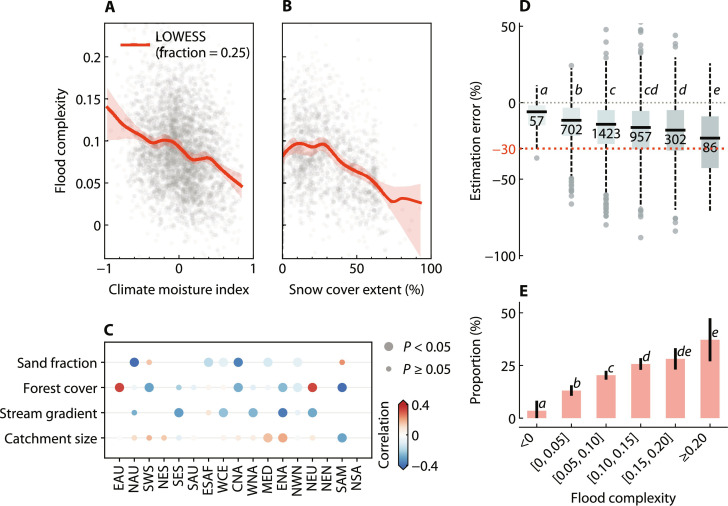
Relationship between flood complexity and catchment attributes, and its impact on estimating large flood magnitudes. (**A** and **B**) The relationship between flood complexity and average CMI (A) and snow cover extent (B) over the catchment, using the locally weighted scatterplot smoothing (LOWESS) of the points and 95% confidence interval from 1000 bootstraps. Only wet catchments (CMI ≥ 0) are considered in (B). (**C**) Spearman’s rank correlations (within each IPCC climate reference region) between flood complexity and catchment size, catchment-average stream gradient, forest cover extent, and sand fraction in soil, where marker size indicates statistical significance and only regions with sufficient attribute variability (e.g., the interdecile range of an attribute in the region is at least 60% of its total interdecile range across all catchments) are shown. (**D**) Estimation error in the magnitude of the largest observed floods per catchment against different levels of flood complexity (*x* axis). The estimation error in each catchment is calculated as the relative error between the estimated magnitude extrapolated from all other flood events and the observed magnitude of the largest flood in the observations (see Materials and Methods). A negative error indicates an underestimation of the largest flood magnitude. Box plots show the median, quartiles, and 1.5× interquartile range. The sample size per bin and statistical significance (one-sided *t* test, α = 0.05) are noted, with shared letters (e.g., *c* in the third and fourth bins) indicating no significant difference in mean errors between bins. (**E**) Proportions of catchments where the largest flood is underestimated by at least 30%, i.e., below the red dashed line in (D). The error bar indicates the 95% confidence interval, which is approximated as $\hat{p}\pm 1.96\sqrt{\frac{\hat{p}\left(100-\hat{p}\right)}{n}}$ ( $\hat{p}$ is the estimated proportion and *n* is the sample size), and letters signify significance between proportions (one-sided *z* test, α = 0.05).

The variation in catchment-specific flood complexity between and within the Intergovernmental Panel on Climate Change (IPCC) reference regions (Fig. 4D) suggests that it may be influenced by climatic conditions, as well as by local differences in catchment characteristics, such as physiography, vegetation, and soil properties. To further investigate the conditions that may promote high flood complexity in a catchment, we compared flood complexity across different catchment attributes (fig. S3). We find that drier catchments and wet catchments with moderate snow cover tend to display high flood complexity (Fig. 5, A and B). Generally, rainfall-runoff processes in humid catchments are assumed to be more linear due to the reduced variability of hydrological conditions, whereas catchments in arid environments may experience more disruptions to within-basin connectivity (*33*–*35*). In wet catchments with moderate snow cover, the potential interactions between both rainfall and snowmelt processes make flood generation mechanisms more heterogeneous and complex than in catchments dominated by rainfall or snowmelt alone. This relationship holds under a more stringent criterion for the predictive performance of the ML models (fig. S11, E and F). Spearman’s rank correlation analysis between flood complexity and representative local characteristics across various climate reference regions suggests that catchments with higher flood complexity tend to exhibit larger size, flatter terrain, reduced forest cover, and lower sand content (Fig. 5C). These associations may be related to the effects of scale and soil storage capacity (*22*, *33*, *36*). For example, with increasing catchment size, the flood generation mechanisms are expected to become more diverse, interactive, and long-lasting, leading to potentially greater heterogeneity in the physical processes underlying floods of different magnitudes (*37*, *38*). However, it should be noted that larger catchments tend to smooth the effects of within-basin process heterogeneities on their discharge responses more than smaller ones (*26*, *34*, *36*). Nevertheless, measurement uncertainty and confounding factors may complicate the correlation analysis for different flood processes, which warrants further investigation.

To evaluate the influence of high flood complexity on the reliability of extreme flood estimates, we assessed the potential errors that may arise when estimating extreme flood magnitudes in catchments with different levels of flood complexity (Materials and Methods). Specifically, we conducted flood frequency analysis based on all annual flood events except the largest one within individual catchments. Our analysis indicates a tendency toward underestimation of the largest flood magnitudes in catchments with higher flood complexity (Fig. 5, D and E). Further experiments with various components of flood frequency analysis, such as different estimation methods and plotting positions (fig. S12), confirm this general pattern, although it should be noted that the magnitude of underestimation varies when different techniques are used. For example, in the 388 catchments with a flood complexity > 0.15 (the green and white points in Fig. 4C), the estimated magnitude of the largest floods is, on average, 17.0 ± 3.5% lower than the observed values across the methods used. This level of underestimation, despite the existing variability, highlights potential risks in practical hydrological design and flood management (*26*, *39*, *40*). Our results suggest that catchments without recorded extreme flood events may experience unexpectedly large events in the future, and such a case could be exacerbated if the physical processes in flood generation in the catchment are heterogeneous due to compounding effects between flood drivers. Note that the observed increase in underestimation error with rising flood complexity is unlikely to be caused by a decrease in the sample size used for flood frequency analysis, as we did not find a negative correlation between flood complexity and sample size [correlation coefficient (*r*) = 0.07]. Sensitivity analyses show that the conclusion is robust even if only catchments with a longer observation period were considered (fig. S13). Nonetheless, given the overall short record lengths of our samples, the implications of flood complexity for practical risk management require further investigation.

## DISCUSSION

Recent literature has increasingly focused on the compounding effects of drivers of river floods, which can potentially improve our understanding of flood extremes under historical conditions and advance predictive capabilities for future flood risks (*21*, *41*, *42*). However, quantifying the role of these compounding effects across different flood magnitudes and catchments has not been attempted so far. On the basis of advanced explainable ML techniques, this study developed an approach to clarify this relationship in a unified framework (Fig. 1). The methodological advance in our study allows detailed dissection and quantification of the intricate interplay of compounding drivers in ways that traditional methods may not fully capture. This opens avenues for studying a variety of weather and climate extremes including river floods, where the role of compounding drivers is particularly important (*15*, *19*). Our results demonstrate that compounding drivers are not only prevalent around the globe (Fig. 2) but are also critical for large river floods in most catchments (Fig. 3). They can exacerbate flood magnitude and lead to extreme flood events even when individual drivers are moderate. The contribution of compounding effects often increases with flood magnitude, but the strength of this increase varies across catchments according to the climatic conditions and local catchment characteristics (Figs. 4 and 5, A to C). Ignoring the substantial variability of the contribution of compounding effects across different flood magnitudes in many catchments could potentially lead to an underestimation of extreme flood hazards (Fig. 5, D and E), which warrants careful consideration in flood infrastructure design and construction. Our results suggest that flood risk assessment and management particularly in arid and snow-rain mixed catchments should be approached with caution.

The origins of large (extreme) floods have been a subject of ongoing debate regarding whether such events are fundamentally distinct from small floods or merely differ in the magnitude of the contributing factors (*25*, *40*, *43*). The clarification of this issue is paramount in selecting and designing appropriate flood estimation methods, which often assume homogeneity in flood samples and are widely used in engineering practice (*24*). However, our findings suggest that this disagreement might be reconciled by considering the catchment conditions that influence the compounding nature of river floods. This is especially relevant in regions with high flood complexity, such as arid and snow-rain mixed catchments, where conventional statistical methods based on the assumption of homogeneity may considerably underestimate the magnitude of extreme floods. Recently, a few alternative approaches have been proposed to explicitly account for diverse flooding mechanisms in flood frequency analysis (*24*, *44*, *45*). We propose further incorporating the concept of flood complexity into hydrological practice to improve flood risk preparedness and assessment.

The ubiquity of compounding drivers in river floods poses a considerable challenge to flood risk management in a warming climate, as the magnitudes and associated probabilities of extreme floods will be affected by the combined effects of the varying trends in different flood drivers (*9*, *14*), thereby challenging current risk management measures. Our study enhances this understanding by quantifying the specific contributions of each driver, providing a solid basis for accurately projecting how changes in individual drivers might affect flood dynamics under climate change, which is critical for developing future flood scenarios and effective adaptation strategies. Moreover, climate change is likely to increase the now low flood complexity in high-latitude regions because the present snowmelt-dominated flood generation will probably become proportionally more affected by rainfall (*46*, *47*). However, these regions may be underprepared for potential flood risk increases because flood magnitudes have decreased in recent decades concurrent with decreasing snowmelt (*13*). Although snowmelt until now has remained the dominant driver in those catchments, the expected increase in precipitation extremes may soon take over (*16*, *48*), potentially leading to unprecedented flood disasters.

## MATERIALS AND METHODS

### Historical observations and simulations

Daily discharge observations in 3527 catchments around the world (Fig. 1C) from 1981 to 2020 were obtained from the Global Runoff Data Center (www.bafg.de/GRDC, accessed 1 August 2022), which consists of discharge records from approximately 10,000 catchments worldwide. The 3527 catchments were selected using the following criteria. First, the catchment should have daily discharge records for at least 20 years during 1981 to 2020. Second, the catchments are larger than 100 km^2^ to encompass at least one grid cell of the meteorological datasets (at ~9-km spatial resolution) and smaller than 100,000 km^2^ based on (*3*, *23*, *49*) to minimize potential complications arising from spatial heterogeneity in overly large catchments. Third, the predictive relationship between hydrometeorological data and identified peak flows should be well captured by the ML model, as an accurate predictive relation is essential in order to derive meaningful information from ML models (*50*). Therefore, the catchments with an average coefficient of determination (*R*^2^) regression score between the predicted and observed peak discharges in the test periods across all replicated cross-validations below 0.3 were excluded.

Global daily precipitation and air temperature from 1979 to 2020 were obtained from the multi-source weighted-ensemble precipitation (MSWEP) and multi-source weather datasets (*51*, *52*), respectively, with a spatial resolution of 0.1°. Daily rainfall, soil moisture storage, and snowpack were estimated by a gridded implementation of the Hydrologiska Byråns Vattenbalansavdelning (HBV) model (*53*) using daily precipitation and temperature. The model has been well calibrated with daily observed discharge from over 4000 catchments worldwide using the same MSWEP precipitation product (*54*). The simulations between 1979 and 1980 were excluded to account for model warm-up processes. The gridded rainfall, temperature, soil moisture, and snowpack were aggregated to individual catchments (fig. S1), with catchment boundaries delineated using an automated outlet relocation algorithm (*55*).

Overall, the simulated spatially aggregated soil moisture and snowpack for each catchment align well with observation-based estimates, which was confirmed through comparisons with several gridded datasets based on satellite or in situ measurements. The validation process involved comparing the catchment-averaged time series of the model with those of other datasets on the grids where the centroid of each catchment is located. To ensure robustness, only catchments with at least 3000 daily remote sensing observations were considered for the remote sensing product analysis. The median Pearson correlation coefficient between the modeled daily soil moisture and the European Space Agency’s Climate Change Initiative for Soil Moisture combined product (v07.1) (*56*, *57*) is 0.55. In addition, the median Pearson correlation coefficients between the modeled daily soil moisture and the SoMo.ml dataset (available in 2000 to 2019 only)—a global soil moisture dataset derived from in situ measurements using ML (*58*)—are 0.70 (0 to 10 cm), 0.77 (10 to 30 cm), and 0.83 (30 to 50 cm), respectively. Last, the median Pearson correlation coefficient between the modeled snowpack and the ESA satellite snow water equivalent dataset (Northern Hemisphere only) (*59*) is 0.73. Moreover, the simulation data would be reconsidered in ML models in terms of their predictive relationship to the discharge observations. Catchments with underperformed predictions due to potentially poor simulation data were excluded.

### Catchment attributes

We chose CMI, snow cover extent, catchment size, stream gradient, forest cover extent, and sand fraction in soil to reflect certain aspects of a catchment’s climatology, physiography, vegetation, and soil attributes, which considers both their representativeness and relevance to flood generation as reported in the literature (*22*, *36*). These catchment attributes (except catchment size) were derived from the well-established HydroATLAS dataset (*60*), which provides a compendium of descriptive hydro-environmental information for all (sub)basins worldwide with nested levels, each representing consistently sized polygons. We used the highest spatial resolution level (level 12), which has a scale of approximately tens of square kilometers. We then derived the relevant attributes for the 3527 catchments in our study using an area-weighted aggregate based on the coverage (fig. S3). Among these attributes, the CMI was calculated from annual precipitation (P) and potential evapotranspiration (PET) using the equation: [CMI = (P / PET) − 1 if P < PET] or [CMI = 1 − (PET / P) if P ≥ PET]. The CMI ranges from −1 to 1, with a higher value indicating wetter conditions. Catchment size was estimated directly from the catchment boundary. Stream gradient refers to the ratio of the slope within the stream reach to the length of the reach.

### Preprocessing of ML samples

The training and testing targets for the ML models used in this study are identifiable discharge peaks, regardless of their extremeness, in the daily discharge series of each catchment (fig. S2). The identification follows the procedure recommended by the guidelines of the US Water Resources Council, which has been widely adopted in many studies (*61*, *62*). First, all local peaks with a minimum distance *T* = 5 days + log(*A*) between neighboring peaks were selected, where *A* is the basin area in square miles and *T* is rounded to an integer. Then, the criterion that the minimum discharge between two consecutive peaks should be less than 75% must be satisfied; otherwise, the smallest peak in such a pair is removed until the condition is fulfilled for all remaining peaks. In total, we identified 1,582,043 discharge peaks for all 3527 catchments, with an average of 12.2 event peaks per year across the catchments. Note that, although only annual maximum discharges are considered in the subsequent analysis, we trained and interpreted the model on all identifiable peak flows for two main reasons. The first is to increase training samples to better capture diverse runoff processes. The second is to provide an appropriate background for the interpretation of ML models (*63*), which serves as a reference point for identifying the contributions of features in the input to the prediction.

We used the time series of daily precipitation and 7-day mean temperature in the last 7 days before the discharge peaks, and soil moisture and snowpack on the day before this 7-day synoptic window as inputs to the ML model (fig. S2). The 7-day time window was chosen according to previous studies (*5*, *6*, *12*). We averaged the temperature within the 7-day synoptic window instead of using the time series within it to simplify the model complexity for better interpretability and to avoid interpretation instability due to autocorrelation between temperatures in the model inputs.

### Training and interpretation of ML models

We used the LightGBM as the ML model, which is based on decision tree algorithms in a gradient boosting framework (*27*). In our preliminary experiments, we compared it with other tree-based ML models (e.g., random forest and XGBoost) and eventually chose LightGBM after accounting for both model performance and efficiency. For each catchment, we implemented repeated fivefold cross-validation on the processed discharge peak samples for model training, evaluation, and interpretation. Specifically, we repeated the fivefold cross-validation process 100 times for each catchment, each time randomly shuffling the sample set to ensure a unique split. This process ensured that each individual data sample was incorporated into various training sets 400 times and subjected to independent evaluation 100 times due to the repeated fivefold cross-validation. Consequently, the interpretation of results in test periods for each sample was derived from a model trained on different training sample sets. Repeated cross-validation can help reduce the randomness and potential bias of the interpretation baseline.

As prior knowledge, we enforced monotonicity constraints on the input features of rainfall, soil moisture, and snowpack in the model, meaning that these features have a monotonically increasing relationship to the discharge response. Our preliminary experiments showed that such constraints improve the predictive performance of the model. We also disabled the interactions between the input features of rainfall and temperature in the model to ensure that the interpretability of the compounding effects is consistent with our domain knowledge. In each training process, the hyperparameters of the LightGBM model were automatically searched by optimizing the model performance on a subset of the corresponding training samples. The candidate hyperparameters are listed in table S1.

The SHAP interaction values (*28*) were used to explain the model outputs in terms of the predictive contributions of the pairwise interactions between input features. This approach, rooted in game theory, allows us to decompose a prediction into individual contributions from each input feature. For each prediction, these contributions are represented in a matrix format, where each matrix element represents the influence of either a single feature or a pair of features on the model output (Fig. 1B). Accordingly, the SHAP interaction values provide information about both the main effects of individual features (shown on the diagonal of the matrix) and the interaction effects between pairs of features (on the off-diagonal). In this study, we calculated SHAP interaction values for all test samples for each of the catchments. Intuitively, the SHAP interaction values explain why the prediction was different from the expected output (i.e., the average of the training targets). As an additive feature attribution method, the SHAP interaction values between feature *i* and all features (including feature *i* itself) sum to the predictive contribution of feature *i*. Therefore, we derived the predictive contribution of different types of variables by aggregating all the interaction effects of the corresponding variables.

### Determining main driving variables and identifying multi-driver floods

For each of the 100 evaluations, we can derive the aggregated contribution of recent rainfall, recent temperature, antecedent soil moisture, and antecedent snowpack (variables are color-coded in Fig. 1B) to every identifiable discharge peak. Using a catchment in Slovakia as an example, the aggregated SHAP values reveal predictive contributions of the four types of variables in the model (fig. S4A). A positive SHAP contribution value of a specific feature indicates its role in increasing the predicted value (in our case, the discharge peak) relative to the model’s average or baseline prediction, whereas a negative contribution does the opposite. The contributions of recent rainfall, soil moisture, and snowpack all scale with their magnitudes, whereas recent temperature only has a positive contribution when the value is around 5°C, implying its role in flooding by inducing snowmelt. The robustness of the contribution pattern is confirmed by the error bars, which indicate the variation across the 100 evaluations.

We then determined a cutoff threshold to distinguish whether the specific driving variable had a considerable contribution. We used the 80th percentile of the aggregated contribution values for all peaks as the threshold, above which the variable is considered a candidate main driving variable. By focusing on annual maximum discharges, we generated a matrix that records the number of exceedances of the threshold in each of the 100 evaluations (fig. S4B). Notably, the results for a British catchment demonstrate a different pattern, as illustrated in fig. S4 (C and D).

For each flood event in the 100 evaluations, we can derive a set of Boolean values (100 elements in this case) that indicate whether the event is associated with a particular driver or combination. We took the majority as the final result and tested its significance using the binomial test. With 100 evaluations and a significance level of 0.01, a driver or a combination with at least 63 exceedances of the respective thresholds is considered to be significantly associated with the corresponding flood event, where the 63 is the minimum number of exceedances required to meet this significance level in the one-sided binomial distribution. Hence, a flood that is associated with multiple candidate main driving variables at least 63 times in the 100 evaluations is regarded as a multi-driver flood. Conversely, a flood that is associated with multiple candidate main driving variables at most 37 times is regarded as a single-driver flood. Note that a single-driver flood may include cases in which the flood is not associated with any main driving variables (threshold for aggregated contribution never exceeded).

### Importance of compounding effects and flood complexity

Similar to the thresholding of aggregated contribution values, we can threshold positive interactions between features in the SHAP interaction values to identify the main interaction effects that considerably contribute to the prediction of discharge peaks. Specifically, the threshold is calculated as the 80th percentile of the positive interaction values between features (including the main effects of the features) across all the samples in the catchment. Note that each sample has 48 potential pair-wise interaction values, given the 10 features used and the fact that the interactions between the seven input features of rainfall and temperature have been disabled in the model. We then defined the relative number of the main interaction effects to all possible interaction effects as the importance of compounding effects for individual flood events (exemplified in fig. S10). In each of the 100 evaluations, we used the simple regression model to fit a slope between the empirical non-exceedance probability and the importance of compounding effects of annual maximum flood events in a catchment. The regression slope, which we defined as the flood complexity of a catchment, indicates the change in the importance of compounding effects for every 1% increase in the non-exceedance probability of floods. We derived 100 slopes and associated *P* values that test whether the corresponding slope is significantly positive. We used the median slope as the final slope across the 100 evaluations and estimated the combined *P* value using Fisher’s method.

### Errors in estimating large flood magnitudes

The estimation error in the magnitude of the largest observed flood in a catchment is calculated as (*Q*_est_ − *Q*_obs_)/*Q*_obs_, where *Q*_obs_ is the magnitude of the largest observed flood during the study period (from 1980 to 2020) and *Q*_est_ is the estimated magnitude based on the flood frequency analysis. Specifically, we first fitted the available annual discharge maxima during the study period (the largest is assumed to be unknown and thus was not included) using the generalized extreme value (GEV) distribution. For the annual maxima, only events in a calendar year with at least 200 days of discharge records were considered. The parameters of the GEV distribution were estimated using the widely used maximum likelihood method. We then calculated *Q*_est_ for the largest flood from the fitted GEV distribution, given the empirical return period of the largest observed flood based on the classical Gumbel plotting position. To assess the robustness of our findings, additional analyses were performed using different estimation methods in the GEV fit, i.e., the method of moments and L-moments (*64*), and different plotting positions, including Cunnane, Bear, Tukey, and an unbiased method that takes into account specific parameters of the GEV distribution and sample size (*65*), the results of which are presented in fig. S12.
