# Supplementary material for: Compounding effects in flood drivers challenge estimates of extreme river floods
Source: Sci Adv. 2024 Mar 27;10(13):eadl4005. doi: 10.1126/sciadv.adl4005 (PMC10971417; doi:10.1126/sciadv.adl4005)
Supplement: Supplementary file 1 — Figs. S1 to S13 Table S1 [file sciadv.adl4005_sm.pdf]

Science Advances
AAAS

Supplementary Materials for

Compounding effects in flood drivers challenge estimates of extreme river floods

Shijie Jiang *et al.*

Corresponding author: Shijie Jiang, sjiang@bgc-jena.mpg.de

Sci. Adv. **10**, eadl4005 (2024)
DOI: 10.1126/sciadv.adl4005

This PDF file includes:

Figs. S1 to S13
Table S1

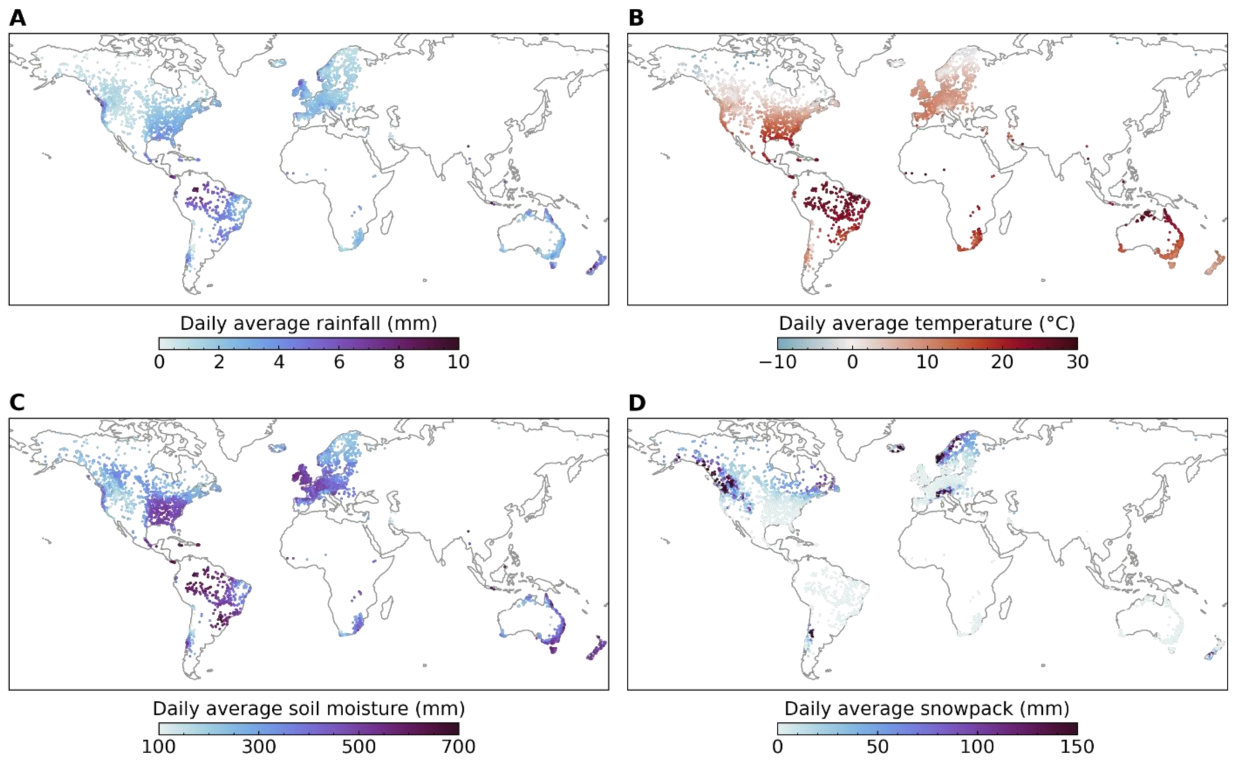

Fig. S1. Hydrometeorological conditions of the 3,527 catchments used in this study. The daily average of rainfall (**A**), temperature (**B**), soil moisture (**C**), and snowpack (**D**) in 1981 to 2020.

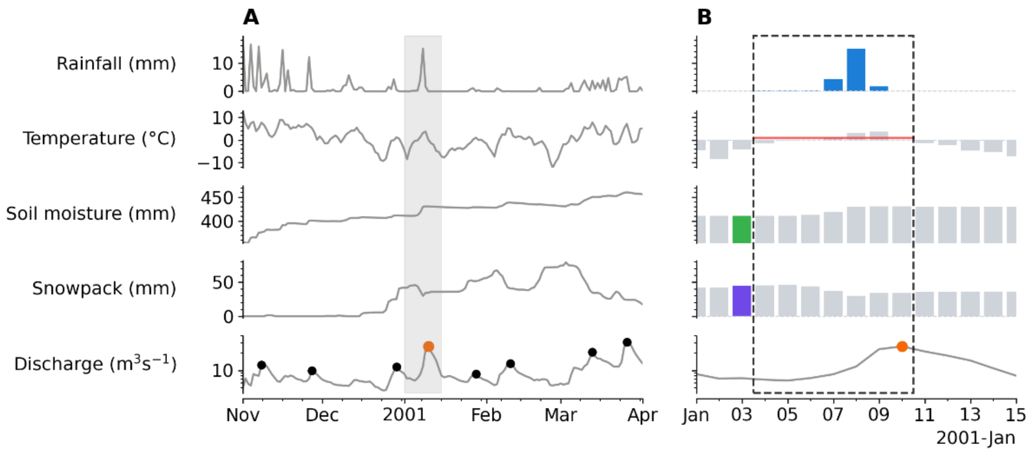

Fig. S2. Illustration of training samples. A catchment in Slovakia with an outlet located at 49.07°N, 18.91°E is used as an example. The solid points in the lower panel in (**A**) show the identifiable discharge peaks used as training targets. The gray shadow (zoomed in panel **B**) highlights the 7-day synoptic window for determining model inputs to the model target indicated by the orange point. In panel B, the colored bars show the input features used; for precipitation, we used the 7 days before the discharge peak; for temperature, we used the average over those 7 days.

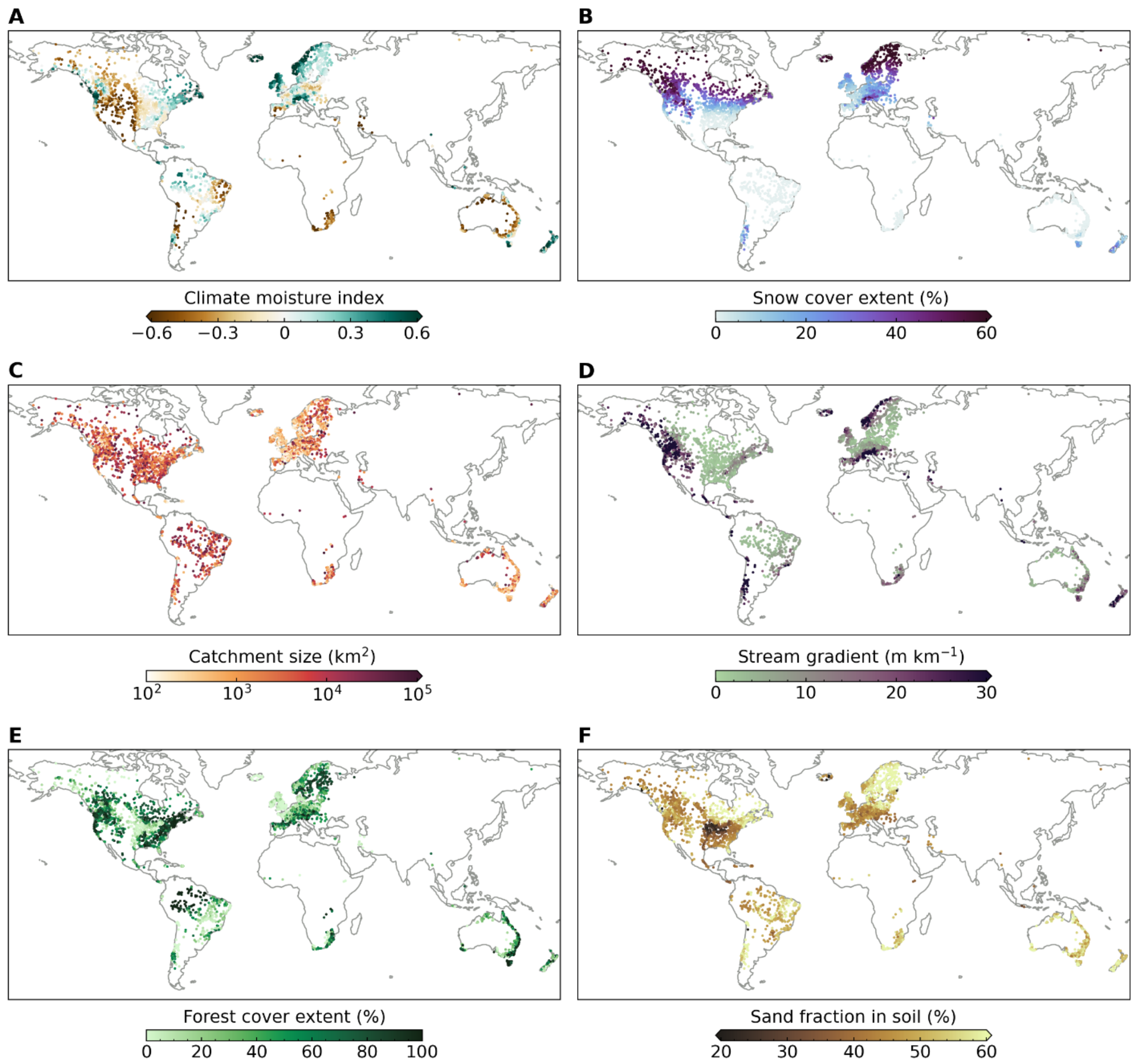

Fig. S3. Climatic and local attributes of the 3,527 catchments used in this study. (**A**) Climate moisture index. (**B**) Snow cover extent. (**C**) Catchment size, which is estimated based on the catchment boundary. (**D**) Stream gradient. (**E**) Forest cover extent. (**F**) Sand fraction in soil.

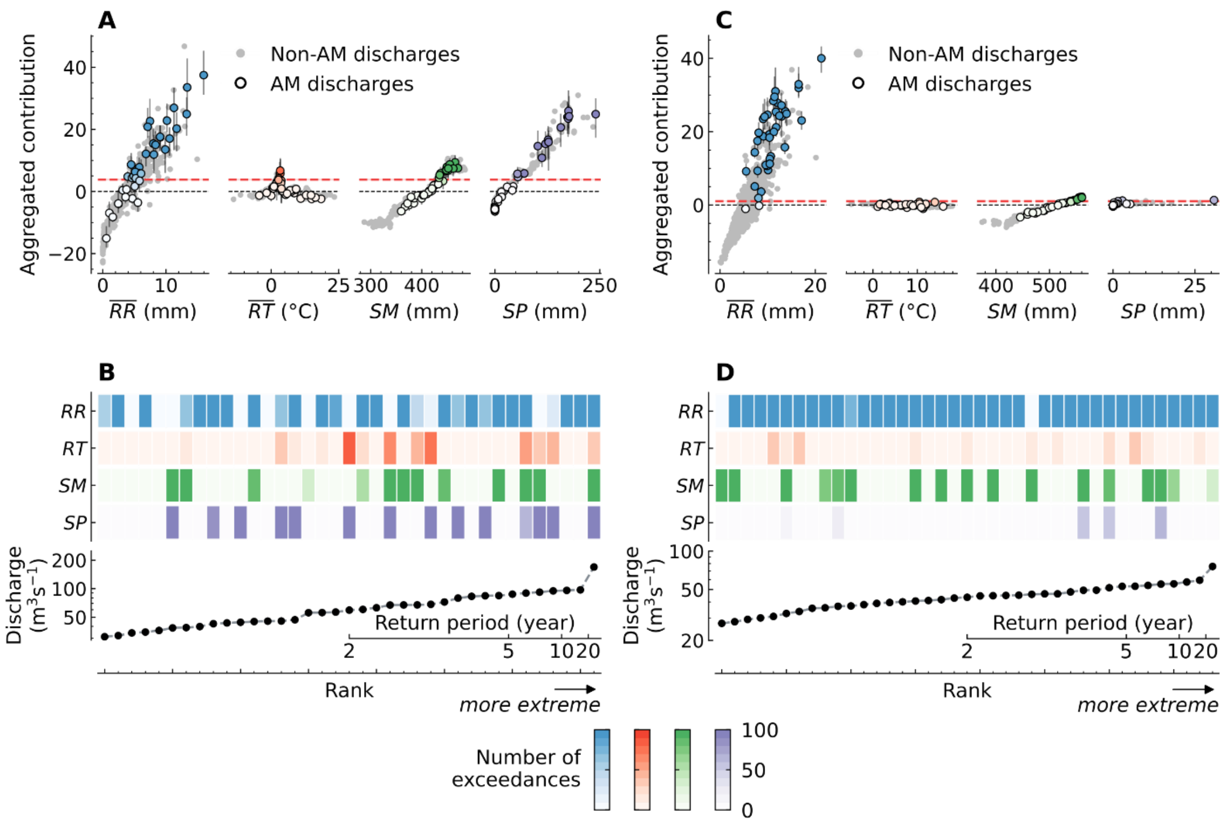

Fig. S4. Procedure for identifying multi-driver river floods. Two catchments, one located in Slovakia with an outlet at 49.07°N, 18.91°E, and another in the United Kingdom with an outlet at 54.44°N, 3.53°W, are used as illustrative examples. (**A**) Relationship between the aggregated contributions and the event-averaged magnitude of different types of variables for all samples in the Slovakian catchment. The points indicate the median of the respective aggregated contributions over 100 evaluations. The error bars indicate the 10th and 90th percentiles of the contribution values for the annual maximum (AM) discharge samples. The red dashed lines indicate the threshold (here, the 80th percentile of the aggregated contributions across all samples) used to identify the candidate main driving variables, above which the variable is considered a considerable contributor. Note the slight variation in the thresholds (overlapped in the figure) across the 100 evaluations. The color saturation of the AM samples indicates the number of exceedances of the corresponding threshold, with higher saturation indicating more exceedances over the 100 evaluations. (**B**) Summary of the number of exceedances of the threshold for the AM samples, ranked according to the magnitude of the annual maximum. The saturation has the same meaning as described in panel (A). (**C** and **D**), Same as panels (A) and (B) but for the British catchment.

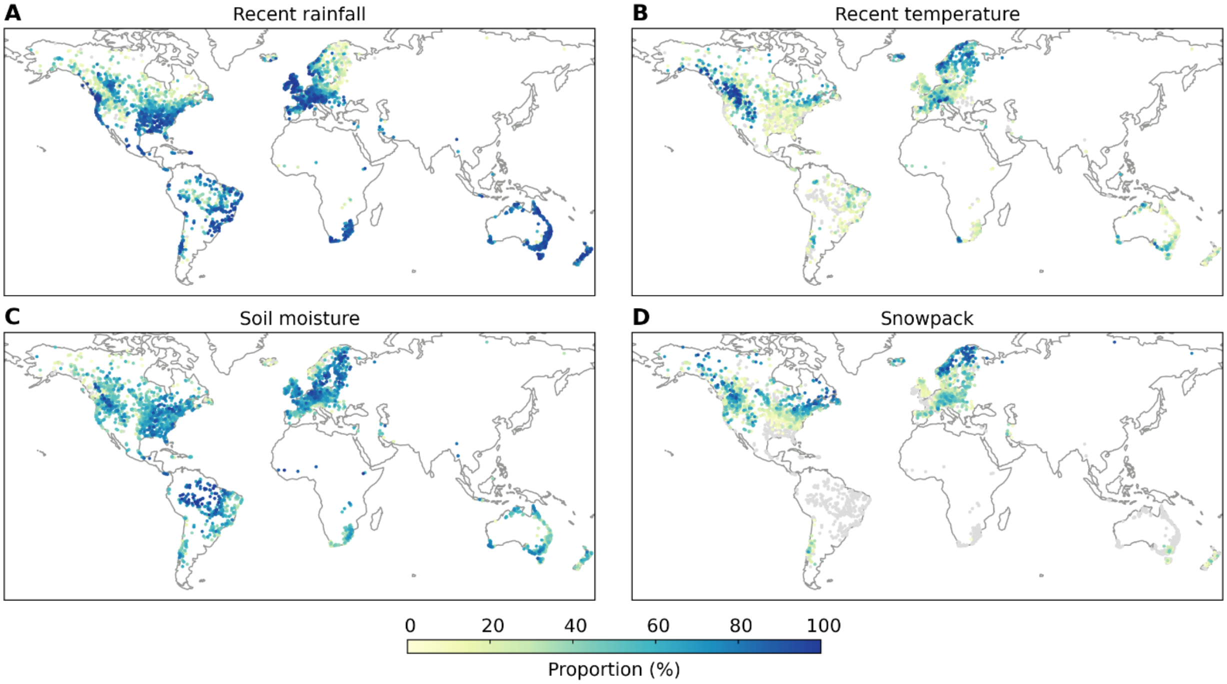

Fig. S5. Main driving variables of annual maximum flood events in the 3,527 catchments. Proportions of recent rainfall (**A**), recent temperature (**B**), soil moisture (**C**), and snowpack (**D**) as main driving variables of annual maximum floods in individual catchments. The gray points indicate catchments for which the respective variable is not considered a main driving variable in any of the considered floods. The 80th percentile of the aggregated contributions of all samples in each evaluation was used as the cutoff for whether the driver contribution was considered (see fig. S4 and **Materials and Methods**).

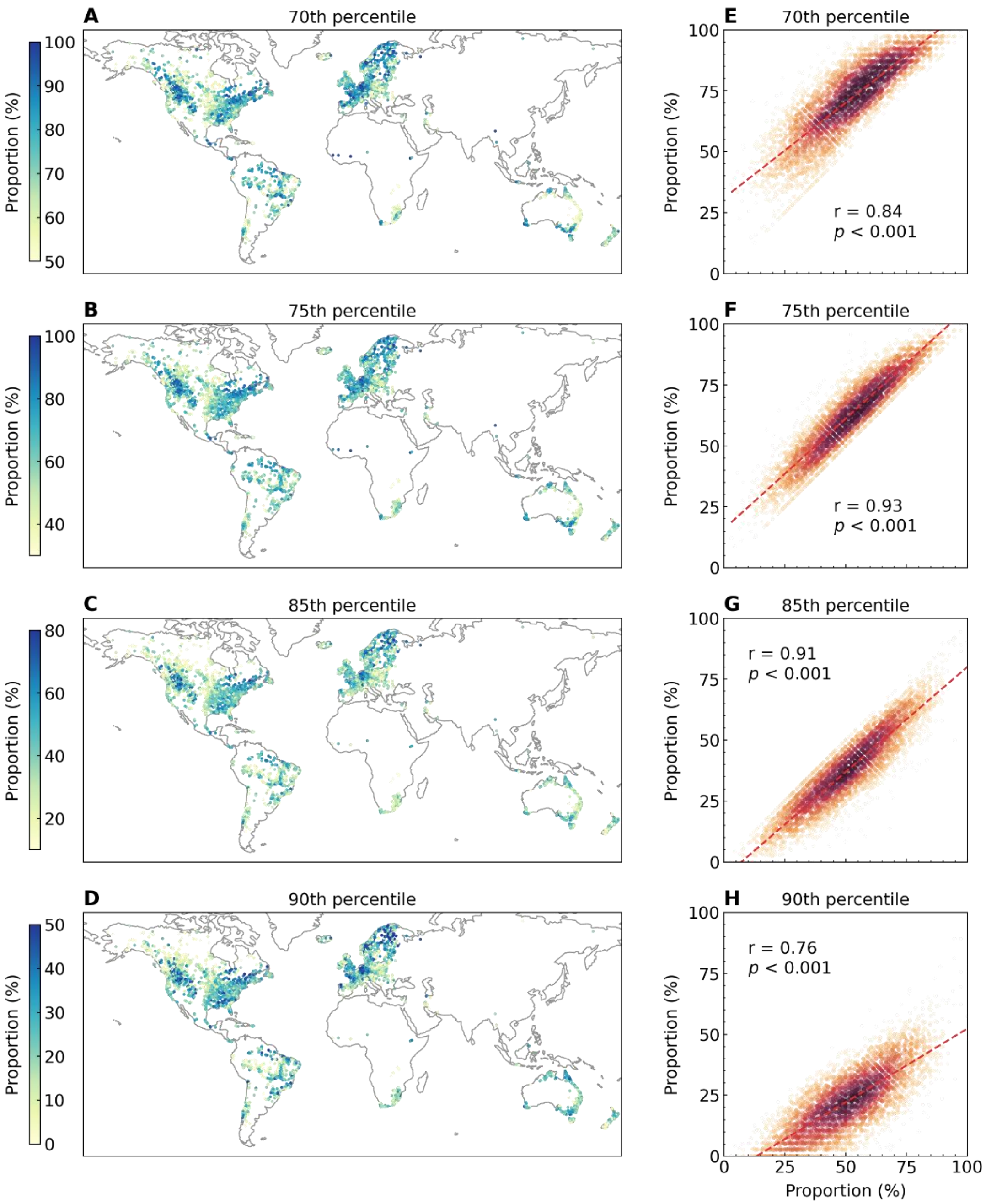

Fig. S6. Sensitivity of using different thresholds to identify main drivers. (**A** to **D**), Proportions of multi-driver floods when using the 70th, 75th, 85th, and 90th percentiles of the aggregated contributions as cutoffs, respectively. (**E** to **H**), Correlations between the proportion of multi-driver floods when using the 80th percentile as the threshold (x-axis) and the proportions when using other thresholds (y-axis).

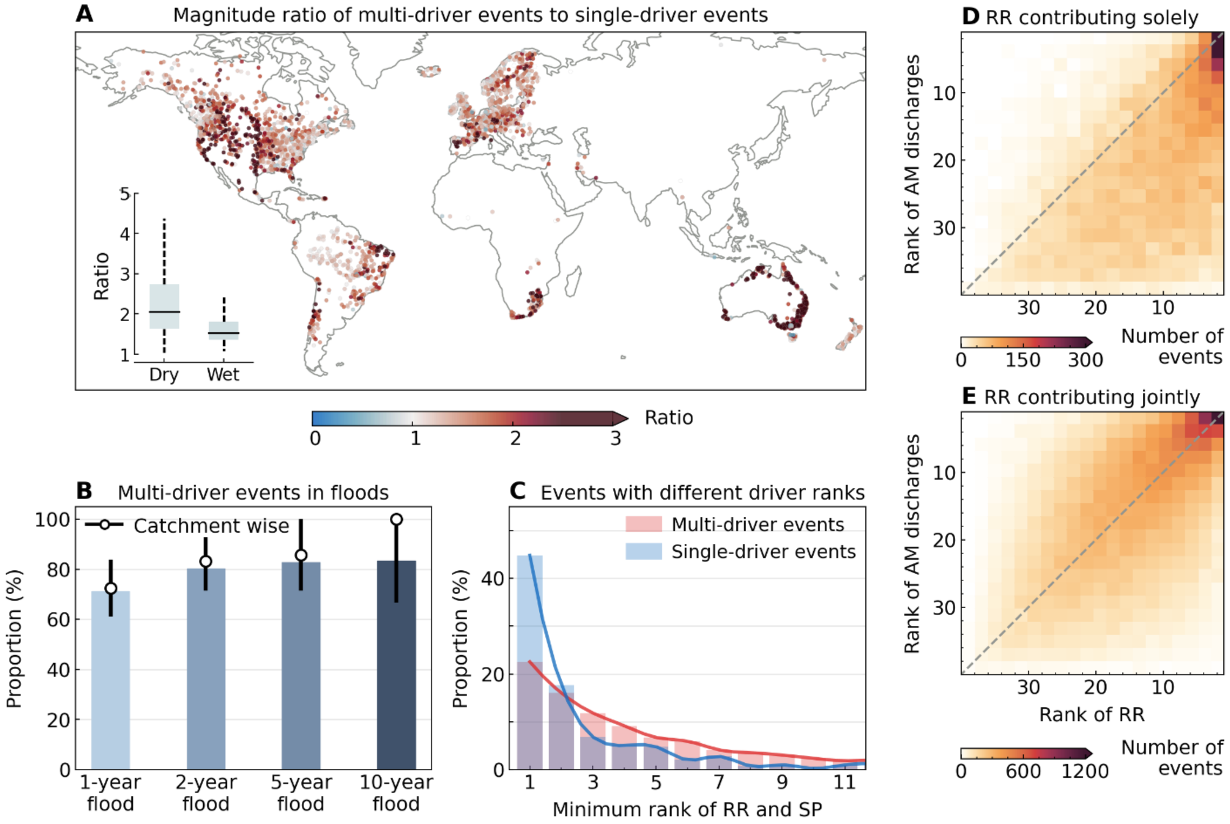

Fig. S7. Impact of compounding drivers on river flood events. (**A** to **E**), Same as Fig. 3, but using the 70th percentile as the threshold to identify main drivers.

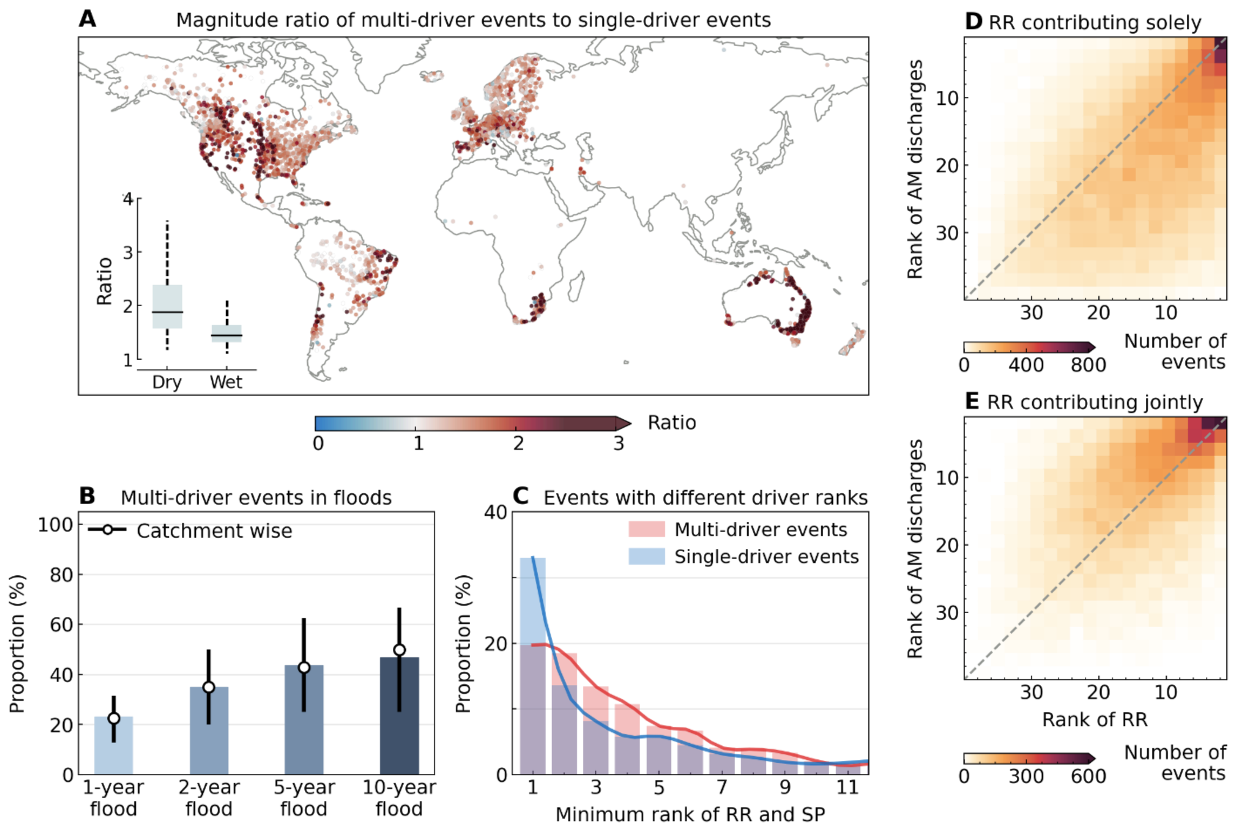

Fig. S8. Impact of compounding drivers on river flood events. (**A** to **E**), Same as Fig. 3, but using the 90th percentile as the threshold to identify main drivers.

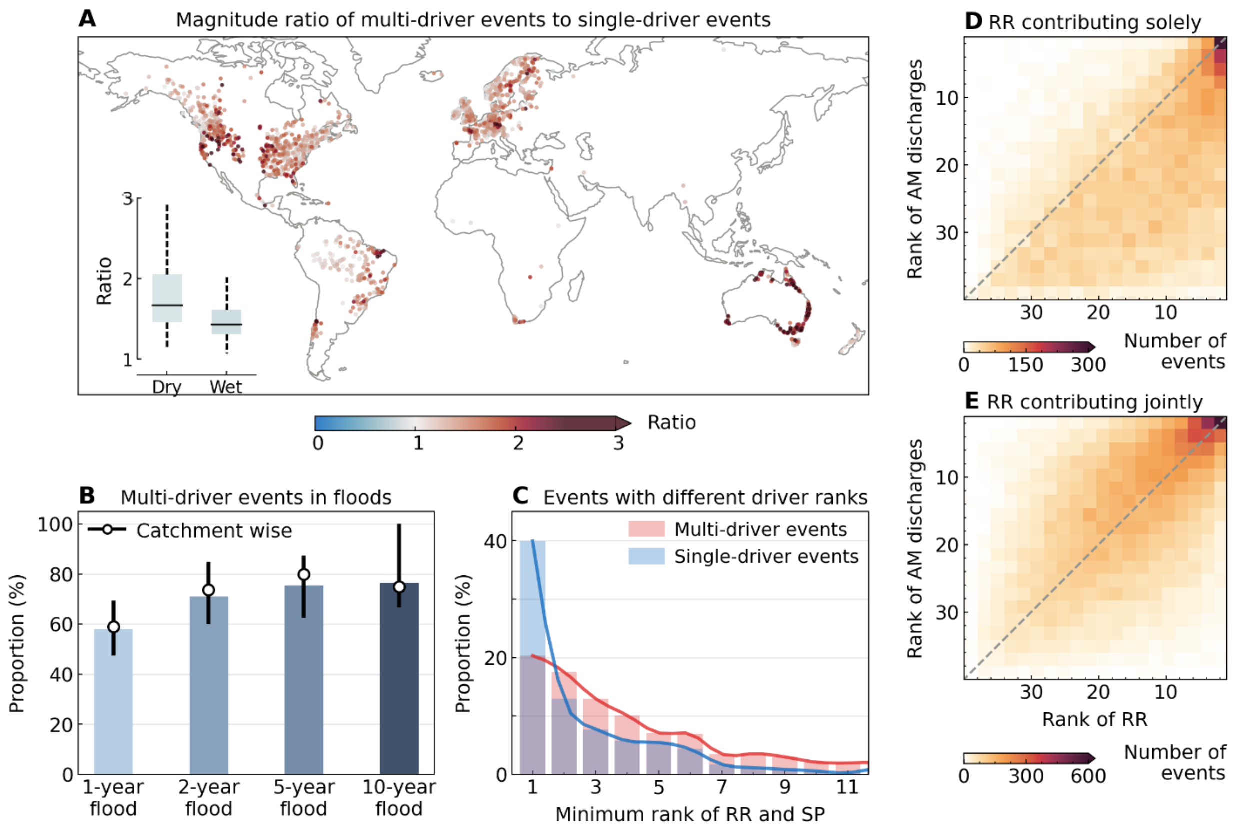

Fig. S9. Impact of compounding drivers on river flood events. (**A** to **E**), Same as Fig. 3, but only 1,886 catchments with the average R^2 regression value in repeated cross-validation greater than 0.6 were considered.

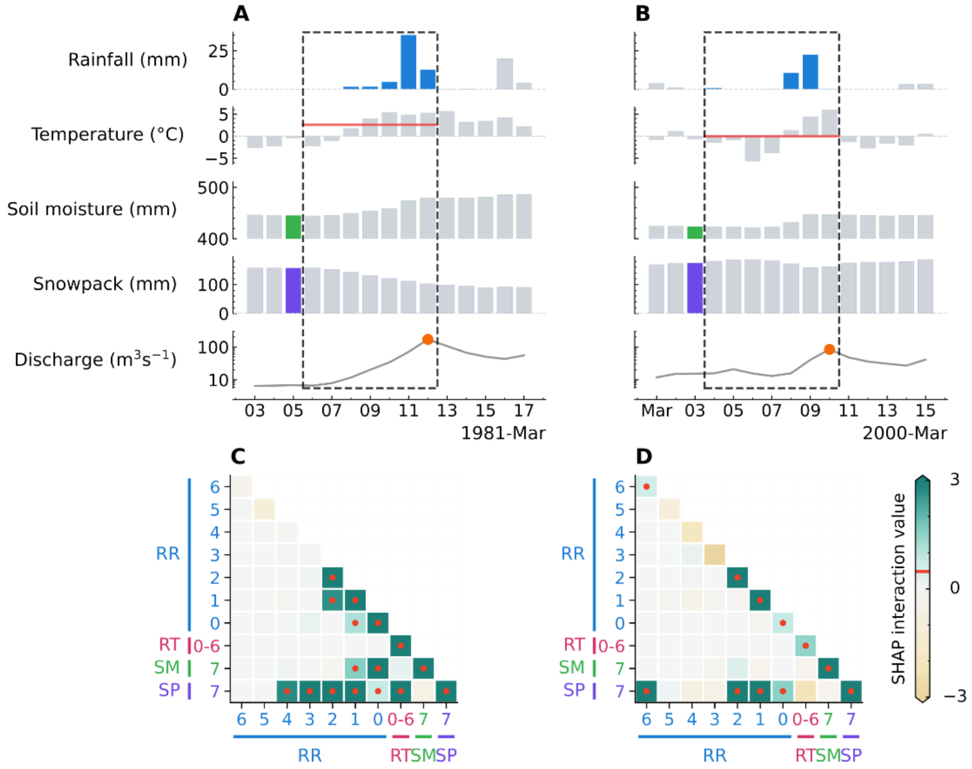

Fig. S10. Illustration of the importance of compounding effects of two flood event samples in one catchment. (**A** and **B**), The input features (colored bars) and model output (orange point) of two flood samples. (**C**) The pairwise interaction effects between features (including the main effects of the features in the diagonal) colored by the SHAP interaction values for the flood sample in (A). The red dots highlight the main interactions where the SHAP interaction value exceeds the threshold (indicated by the red line in the color bar). Here, the threshold is calculated as the 80th percentile of the positive interaction values between features (including the main effects of the features) across all the samples in the catchment. In this case, the number of main interactions is 16, so the importance of compounding effects is 16/48 = 33.3%, where 48 is the number of all potential interactions in the model (note that we have disabled the interactions between the input features of rainfall and temperature in the model). (**D**) The pairwise interaction effects between features for the flood sample in (B), for which the importance of compounding effects is 22.9%.

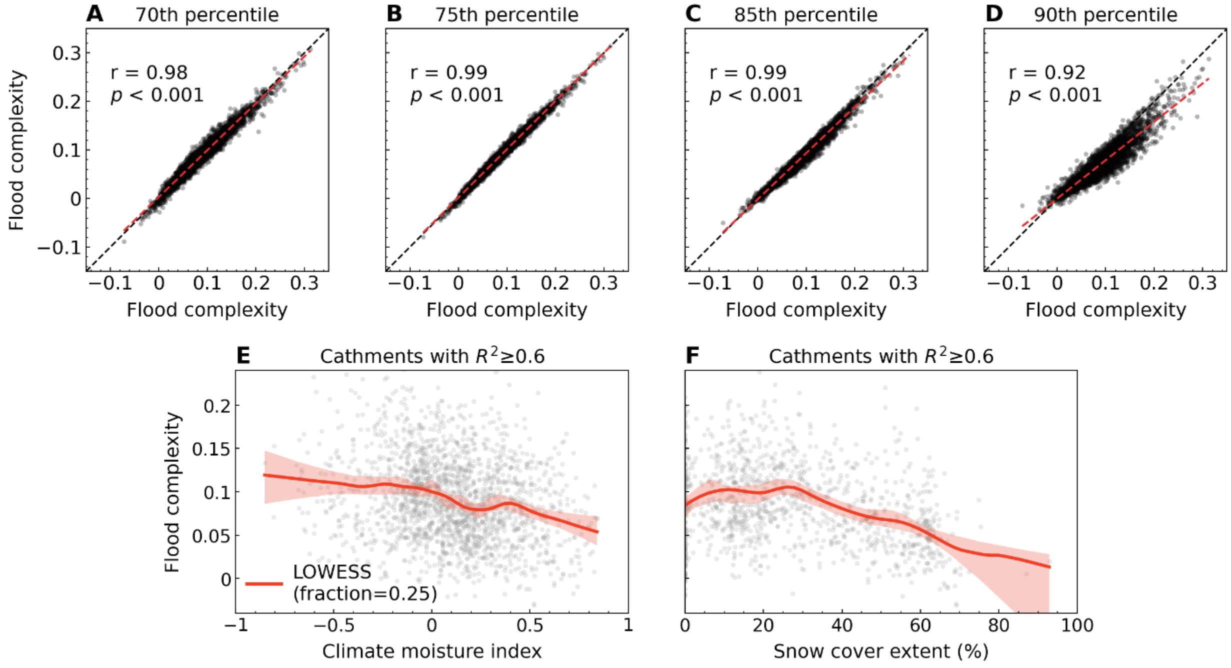

Fig. S11. Sensitivity of using different thresholds to identify main interaction effects and a stricter criterion for the predictive performance of ML algorithms. (**A** to **D**), Correlations between the flood complexity of catchments when using the 80th percentile as the threshold (x-axis) and the proportions when using other thresholds (y-axis). (**E** and **F**), Same as Fig. 5(A and B), but only 1,886 catchments with the average R^2 regression value in repeated cross-validation greater than 0.6 were considered.

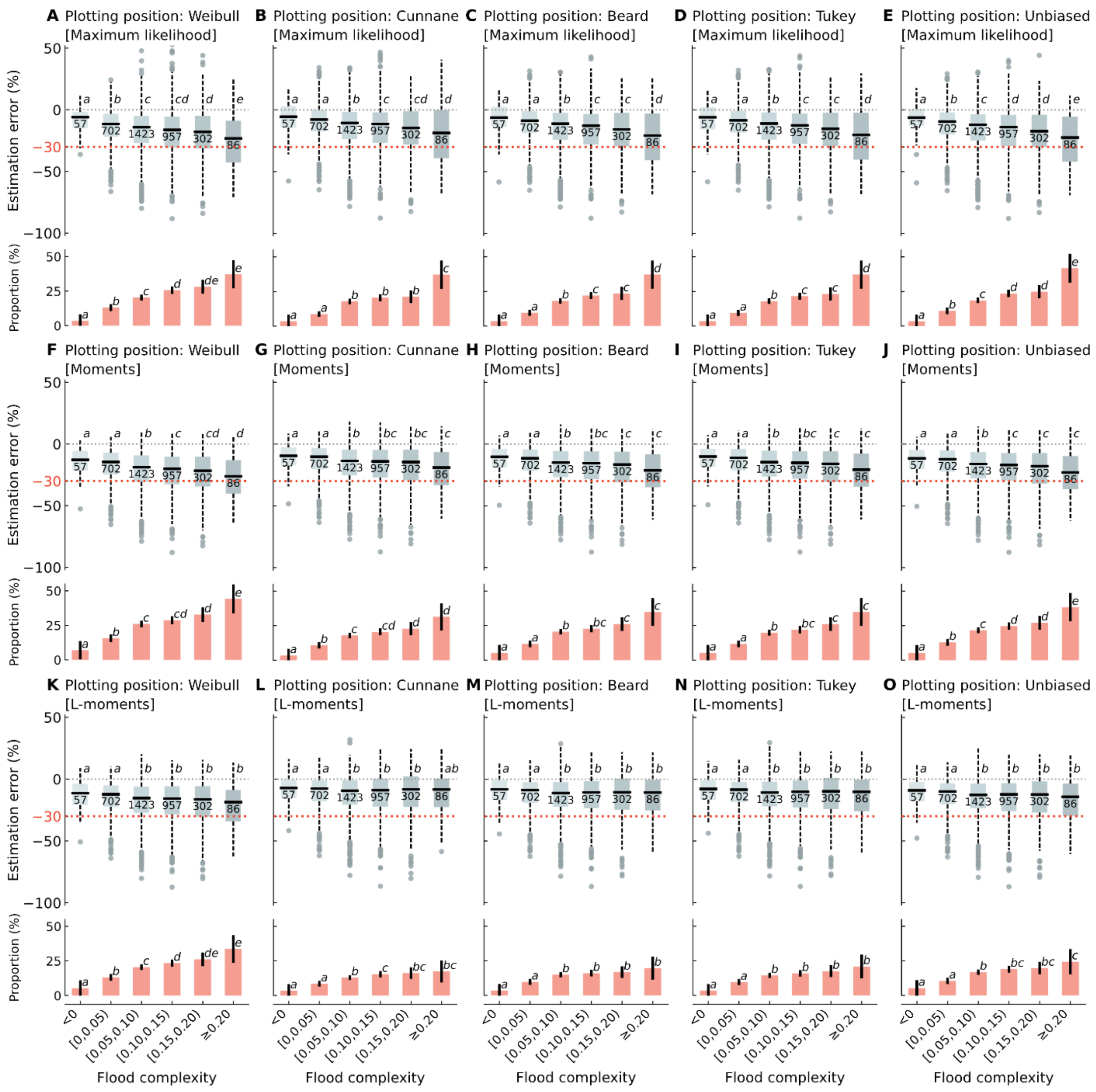

Fig. S12. Impact of flood complexity on estimating large flood magnitudes. (**A** to **O**) parallels Fig. 5(D and E) but compares different estimation methods for fitting the Generalized Extreme Value (GEV) distribution (shown in the rows) and different plotting positions for determining the empirical return period of the largest observed flood (shown in the columns). Specifically, we included the method of moment and L-Moments in addition to the Maximum Likelihood method used in the main text. For plot positions, we considered the Cunnane, Bear, Tukey, and an unbiased method that takes into account both specific parameters of the GEV distribution and sample size (*65*), in addition to the Gumbel method used in the main text. The proportions in these bar plots indicate catchments where the largest flood is underestimated by at least 30%, i.e. below the red dashed line in the box plots.

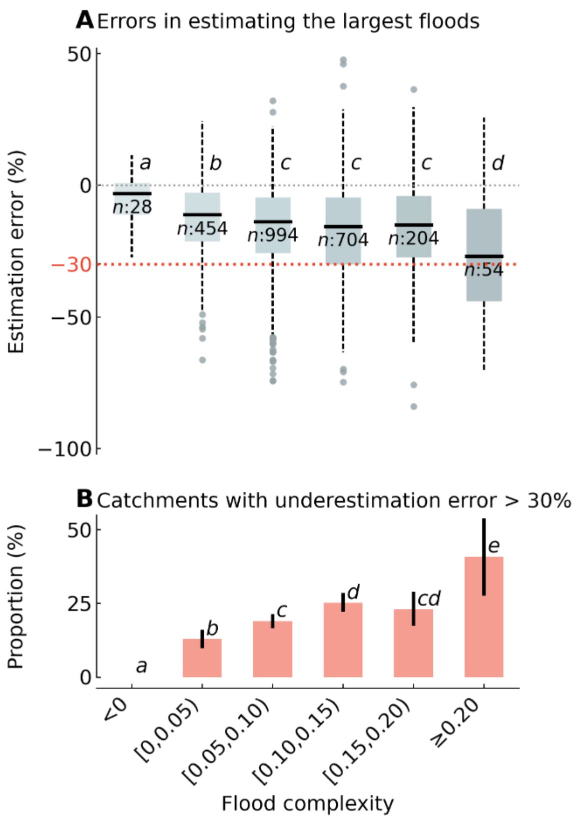

Fig. S13. Impact of flood complexity on estimating large flood magnitudes. Same as Fig. 5(D and E), but only 2,438 catchments with at least 35 observations of annual maximum discharge were considered.

Table S1. Candidate values for hyperparameters of the LightGBM models

The hyperparameters to be determined	The meaning of the hyperparameters	Candidate values
learning_rate	Learning rate	[0.01, 0.02, 0.03, 0.04, 0.05]
n_estimators	The number of boosting iterations	[50, 100, 150, 200]
subsample	The fraction of data to be used for each iteration (tree)	[0.3, 0.5, 0.7]
colsample_bytree	The fraction of features on each iteration (tree)	[0.3, 0.5, 0.7]
max_bin	The max number of bins that feature values will be bucketed in	[8, 16, 24, 32, 64]
min_child_samples	The minimal number of data in one leaf	[3, 5, 7]
